# Effect of topical honey application along with intralesional injection of glucantime in the treatment of cutaneous leishmaniasis

**DOI:** 10.1186/1472-6882-7-13

**Published:** 2007-04-27

**Authors:** Mohammad Ali Nilforoushzadeh, Fariba Jaffary, Shahram Moradi, Roya Derakhshan, Elaheh Haftbaradaran

**Affiliations:** 1 Skin Disease and Leishmaniasis Research Center (Sedigheh Tahereh). Isfahan University of Medical Sciences, Isfahan, Iran; 2 Department of Pharmacology, Isfahan University of Medical Sciences, Isfahan, Iran; 3 Department of Anesthesiology, Pharmacology and Therapeutics, University of British Columbia (UBC), Vancouver, Canada

## Abstract

**Background:**

Leishmaniasis is an endemic disease in Iran. Although many treatments have been suggested for this disease, there hasn't been an effective and safe treatment yet. Regarding the healing effect of honey in the chronic ulcers and its reported therapeutic effect in cutaneous leishmaniasis, we performed a study to better evaluate the efficacy of honey in  cutaneous leishmaniasis and its final scar.

**Methods:**

In a prospective clinical trial, 100 patients with confirmed cutaneous leishmaniasis were selected and randomized into 2 groups. Group A were treated with topical honey twice daily along with intralesional injection of glucantime once weekly until complete healing of the ulcer or for maximum of 6 weeks. Group B were treated with intralesional injection of  glucantime alone until complete healing of the ulcer or for a maximum of 6 weeks, too. The patients were followed for 4 months. The collected data were analyzed statistically using statistical tests including Chi-square, Mann Whitney and Kaplan – Mayer tests.

**Results:**

In this study, 45 patients that had cutaneous leishmaniasis were treated with intralesional glucantime alone and 45 patients were treated with topical honey and glucantime . Ten patients left out the study. In the glucantime alone treated group, 32 patients (71.1%) had complete cure whereas in the group treated with both glucantime & topical honey, 23 patients (51.1%) achieved complete cure. This difference was significant statistically (p = 0.04).

**Conclusion:**

Further studies to better clarify the efficacy of honey in cutaneous leishmaniasis is needed. We suggest that in another study, the efficacy of honey with standardized level of antibacterial activity is evaluated against cutaneous leishmaniasis.

## Background

Cutaneous leishmaniasis is still a large world problem [1]. Iran is one of the 7 important foci of leishmaniasis and Esfahan is one of the most important hyperendemic foci as annually 10–20 thousands of new cases of leishmaniasis are reported [2,3]. Many investigations are performed to find an effective, safe treatment for leishmaniasis. Pentavalent antimonials are still the mainstay of treating all forms of leishmaniasis. The most commonly used organic compounds of antimony are sodium antimony gluconate (SAG) and meglumine antimoniate (MA).

Although the precise mechanism of action is not fully known, the antimonials are known to inhibit glycotic enzymes and fatty acid oxidation in leishmania amastigotes, and there is a dose dependent inhibition in net formation of adenosine triphosphate(ATP) and guanosine triphosphate (GTP)[4].

Honey has been suggested as an effective healing agent for various kinds infected ulcers in both traditional and modern medicine [5]. Topical application of honey has been shown to be effective in treatment of the post-operative wound infections, reducing the need for antibiotics and finally reducing remaining scar[6]. There is a massive accumulation of collagen in the scar tissue but investigations in the embryonic ulcers that healed without scar have shown that collagen organization plays a more important role in the development of the scar than collagen deficiency.

Honey is effective in wound healing through improvement of granulation and epithelializition stages, improvement of debridment and reduction of wound malodor [6-11]. Studies have shown that honey produced from flowers in the Australia and New Zealand (leptospermum species) have antibacterial properties [11-15]. In some reports, honey has antileishmania and anti rubella virus activity [16,17]. In addition, topical hot honey has been used as a traditional treatment in the endemic areas [18].

As  leishmaniasis is a chronic, long lasting ulcer and there is high probability of secondary infection, we designed the following study to evaluate the adjuvant efficacy of the topical honey along with glucantime in the treatment of the cutaneous leishmaniasis.

## Methods

This study was a controlled randomized clinical trial study. Overall, 100 patients with confirmed cutaneous leishmaniasis were evaluated. This study was performed in Skin Disease and Leishnaniasis Research center. The study was approved by the ethic committee of the Skin Disease and Leishnaniasis Research center (SEC. 84210). The patients were randomized into 2 groups, using Random allocation software. (ver 1.0, may 2004; Saghaei)

The inclusion criteria for the patients were: confirmed cutaneous leishmaniasis with direct smear, no history of systemic or topical therapy for cutaneous leishmaniasis, absence of the malnutrition or severe predisposing disease such as cardiac, renal or hepatic disease and other contraindication for glucantime.

Selected patients were in the age range of 7–70 yrs old. Pregnant and lactating women were excluded. The lesions should not be more than 3 months old and the patients should not be treated with the drugs that had interaction with glucantime.

After giving enough information to the patients, informed consent were taken from them.

Patients were randomized into 2 groups. Group A were treated with intralesional injection of the glucantime and with honey soaked gauze. The lesions were firmly dressed with the honey soaked gauze twice daily. Intralesional injection of meglumine antimoniate (glucantime) was performed once weekly until complete healing of the ulcer or for maximum of 6 weeks. Intralesional meglumine was administered enough to blanch the lesion and 1 mm rim of the surrounding normal skin. Group B were treated with intralesional injection of the glucantime alone until complete healing of the ulcer or for maximum of 6 weeks.

Complete healing in was defined as disappearance of the induration and complete reepithelization of the ulcer. Patients were followed weekly for 6 consecutive weeks and at the end of the 2^nd^, 3rd and, 4^th ^month.

If the patients had not acheived complete healing after 6 weeks of the treatment, direct smear and culture were performed again. Diameter of the lesion and size of the erythema, induration and ulcer were measured by use of the millimeter papers. These evaluation performed by the investigators who were blinded to the type of treatment. In the case of recurrence, parasitology exam was performed. All of the side effects and response to treatment were recorded.

At the end of treatment and follow up, response to treatment was defined as:

1- Complete healing of the lesions was defined as complete clinical and parasitological healing (negative direct smear).

2- Partial healing of the lesions was defined as the decrease of the size and indurations of the lesions.

3- Non-responsive was defined as no clinical change or progression of the lesions.

The collected data were analyzed using SPSS 10 software and statistical tests including Chi-square, Mann Whitney and Kaplan – Mayer.

## Results

Demographic characteristics of the patients are shown in table 1. There was no significant difference regarding demographic characteristics between the 2 groups.

**Table 1:** Demographic Characteristics of the groups

**Parameters**	**Sub group**	**Honey + meglumin Antimoniate**	**Meglumin Antimoniate**	**P value**
**Sex n (%)**	**Male**	33(73.3%)	28(62.2%)	0.18
	**Female**	12(26.7%)	17(37.8%)	
**Age** **Mean ± SD**		26.1 ± 15.1	25.6 ± 14.9	0.89
Location of the lesions* n (%)	**Foot**	26(57.8%)	16(35.6%)	0.057
	**Hand**	16 (35.6%)	21 (46.7%)	0.39
	**Other areas**	3(3.3%)	8(8.9%)	0.19
**Number of the lesions** **Mean ± SD**	**Total**	1.3 ± 0.7	1.7 ± 0.75	0. 016
	**Foot**	1.46 ± 0.85	1.5 ± 0.89	0.82
	**Hand**	1.18 ± 0.4	1.95 ± 0.66	0.001
	**Other areas**	-	1.5 ± 0.53	-
Type of the lesions**	**Plaque**	27 (60%)	25(55.6%)	0.8
**n (%)**	**Crusted plaque**	6(13.3%)	4(8.9%)	0.7
	**Nodule**	7(15.6%)	10(22.2%)	0.59
	**Papule**	5(11.1%)	2(4.4%)	0.43
	**Ulcerated plaque**	0(0%)	4(8.9%)	0.11

*There was no significant difference between two groups regarding location of the lesions (p = 0.07)

** There was no significant difference between two groups regarding type of the lesions (p = 0.17)

In the glucantime alone treated group, 32 patients (71.1%) had complete cure where as in the group treated with both glucantime & topical honey, 23 patients (51.1%) achieved complete cure. This difference was significant statistically (p = 0.04).

**Figure 1:**
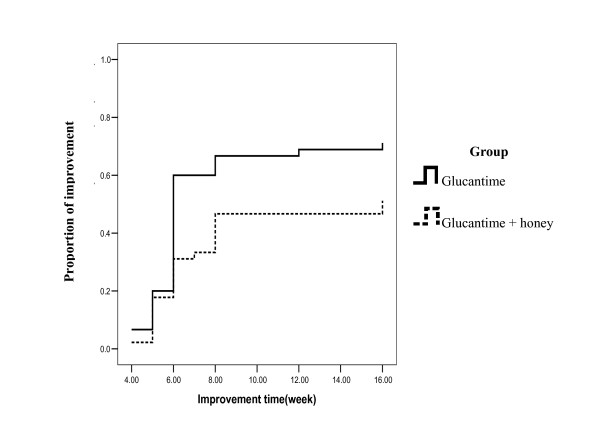
Comparison between healing of the leishmaniasis cutaneous ulcer in the two groups.

Figure 1 shows the healing trend in the evaluated patients. Overall, in the topical honey treated group, 13 patients left out the study. One patient (7.7%) left out the study because of contact dermatitis to honey and 12 patients left out of the study because of progression of their lesions. In the glucantime treated group, 10 patients left out the study because of progression of their lesions. There was no significant difference between 2 groups regarding progression of lesions (P= 0.7). Mean of healing time in the honey and glucantime group after omitting the exited patients were 7.04 ± 3.09 and 6.3 ± 2.29 weeks, respectively but this difference was not significant (P = 0.3).

There was no significant relation between the number of lesions and response to treatment in the 2 groups.

## Discussion

Cutaneous leishmaniasis is an endemic disease in many countries including Iran. Side effects of the antimony compounds that are first line of treatments have increased the tendency for use of drugs with herbal and animal origin to treat this disease.

Honey is used as a healing agent for infected ulcers both in the modern & traditional medicine. It is used as an effective dressing for wounds, burns and scratches to reduce edema, inflammation and pain [6]. The antibacterial activity of honey is primarily due to hydrogen peroxide generated by the action of an enzyme that the bees add to the nectar, but there are some floral sources that provide additional anti bacterial components. We used honey in combination with intralesional glucantime for treatment of the cutaneous leishmaniasis. The honey was held on the lesions for enough time to have a more efficacy. However, our study showed that when honey used as an adjuvant therapy with intralesional glucantime, the effect of glucantime in healing of the leishmaniasis ulcer was actually decreased. In fact, patients who were treated with this combination treatment had less improvement in their lesions as compared with intralesional glucantime, alone. Our finding is not explainable logically as honey has anti inflammatory properties and provides nutritional supply for the damaged tissue [7,9]. It is possible that honey is diluted by serum exuding from wounds and therefore decreased its therapeutic efficacy. In addition, this finding may be due to drug interaction and prevention of the bioavailability of the glucantime by honey or vice versa. Our results showed that combination therapy with glucantime and honey can not be considered as effective treatment for cutaneous leishmaniasis in spite of the therapeutic effects of the honey in skin lesions.

## Conclusion

Further studies to better clarify the efficacy of honey in cutaneous leishmaniasis is needed. Several brands of honey with standardized level of antibacterial activity are commercially available in Australia and New Zealand. We suggest that in another study, the efficacy of these types of honey are evaluated against cutaneous leishmaniasis.

## Competing interests

The author(s) declare that they have no competing interests.

## Authors' contributions

MAN performed scientific review. FJ performed scientific review and statistical analysis. SM prepared honey. RD performed statistical analysis. EH carried out treatment of patients and prepared the manuscript.

## Pre-publication history

The pre-publication history for this paper can be accessed here:


